# Case Report: Novel RPGRIP1L Gene Mutations Identified by Whole Exome Sequencing in a Patient With Multiple Primary Tumors

**DOI:** 10.3389/fgene.2021.620472

**Published:** 2021-02-01

**Authors:** Jiani Guo, Yu Yang, Zhuqing Ji, Mengchu Yao, Xiaotian Xia, Xiaofeng Sha, Mingde Huang

**Affiliations:** ^1^Department of Medical Oncology, The Affiliated Huaian No.1 People's Hospital of Nanjing Medical University, Huai'an, China; ^2^Department of Medical Oncology, Huai'an Hongze District People's Hospital, Huai'an, China

**Keywords:** RPGRIP1L, multiple primary tumors, somatic mutation, pancreatic adenocarcinoma, whole-exome sequencing

## Abstract

A 78 years old Chinese woman with five different cancer types and a family history of malignancy was the subject of this study. Pancreatic adenocarcinoma and gingival squamous cell carcinoma tissues were obtained from the patient and sequenced using Whole Exome Sequencing. Whole exome sequencing identified 20 mutation sites in six candidate genes. Sanger Sequencing was used for further validation. The results verified six mutations in three genes, OBSCN, TTN, and RPGRIP1L, in at least one cancer type. Immunohistochemistry was used to verify protein expression. mRNA expression analysis using The Cancer Genome Atlas database revealed that RPGRIP1L was highly expressed in several cancer types, especially in pancreatic adenocarcinoma, and correlated with patient survival and sensitivity to paclitaxel, probably through the TGF-β signaling pathway. The newly identified somatic mutations in RPGRIP1L might contribute to pathogenesis in the patients. Protein conformation simulation demonstrated that the alterations had caused the binding pocket at position 708 to change from concave to convex, which could restrict contraction and extension, and interfere with the physiological function of the protein. Further studies are required to determine the implication of RPGRIP1L in this family and in multiple primary tumors.

## Introduction

Multiple primary tumors (MPTs) are a phenomenon rarely clinically seen. MPTs are defined as two or more malignancies simultaneously or successively occurring in an individual (Vogt et al., 2017). The incidence of MPTs is higher in the elderly, especially in people aged 50–60 (Patrascu et al., 2010). Despite understanding that multiple factors including unhealthy lifestyle (Hori et al., 2011), chemoradiotherapy (Oeffinger et al., 2013), and genetic mutations (Park et al., 2014), are involved in the development of MPTs, the pathogenesis of MPTs remains unclear. Deleterious germline mutations and defects in DNA repair genes are closely related to MPTs (Tiwari et al., 2016). Recently, Xu et al. identified a homozygous germline insertion mutation in *WWOX*, a DNA repair-related gene, from a 26 years old female patient with MPTs (Xu et al., 2019). Nevertheless, further investigation is required to clarify the molecular mechanisms underlying MPTs.

## Materials and Methods

### Clinical Information and Samples in the Study

The patient is a 78 years old Chinese woman, who had successively developed five different cancer types. This patient developed hepatocellular carcinoma in 2000 (for which she received interventional therapy in the liver), colorectal adenocarcinoma in 2002 (for which she received a partial colon resection), invasive breast carcinoma in 2011 (for which she received radical mastectomy of the left breast), gingival squamous cell carcinoma in 2018 (for which she received right side gingival tumor resection), and pancreatic adenocarcinoma in 2018 (for which she received a partial pancreatectomy). Based on the patients' report, none of the cancers were treated with radiotherapy or chemotherapy. The patient was admitted with a right cervical mass in November 2019 and mass biopsy was performed in The Affiliated Huaian No.1 People's Hospital of Nanjing Medical University. The pathology results showed metastatic squamous cell carcinoma that had originated from the gingival tumor and the patient received chemoradiotherapy in our hospital. Moreover, the patient had a very complicated family history of malignancy, which showed that the patient's mother died of esophageal squamous cell carcinoma, and her two brothers (the patient's uncles) also died of malignancies (with no exact details). The patient had two older sisters, two older brothers, and a younger brother. Her oldest brother died of leukemia, and her second oldest brother, who had a history of cardia carcinoma, was alive. Her younger brother died of cardia carcinoma and had a history of gingival squamous cell carcinoma. However, the rest of her family, including her father, two sisters, and her four children, had no cancer history (Supplementary Figure 1). Therefore, it is valuable to discuss the etiology and pathogenesis of this case. This study was approved by the Clinical Research Ethics Committee of The First Affiliated Hospital of University of Science and Technology of China (Protocol number: P-015). The patient and her family members provided written informed consent for study participation. A written, informed consent was obtained from the participant for the publication of this case report. Our study followed the institutional ethical guidelines approved by The Affiliated Huaian No.1 People's Hospital of Nanjing Medical University (Huai'an, China).

### DNA Extraction

Sample loss and damage meant that only two cancer samples (gingival and pancreatic) were available for further sequencing. The patient's blocks of gingival squamous cell carcinoma and pancreatic adenocarcinoma after resection, as well as her peripheral blood samples were collected. Genomic DNA of the samples was isolated using the FastPure FFPE DNA Isolation Kit (Vazyme, Nanjing, China), and purified using 1% Sepharose electrophoresis. DNA purity and concentration were measured using a NanoPhotometer® spectrophotometer (IMPLEN, CA, USA) and Qubit® 3.0 Flurometer (Life Technologies, CA, USA).

### Whole Exome Sequencing and Data Analysis

To prepare the Illumina sequencing libraries, the SureSelect Human All Exon kit V6 (Agilent Technologies, Santa Clara, CA, USA) was used. Genomic DNA samples (3 μg) were randomly fragmented into 150–200 bp fragments using a Covaris S2 sonicator (Covaris, Woburn, MA, USA) and subjected to library preparation according to the Sure Select XT Target Enrichment System (Agilent Technologies, Santa Clara, CA, USA). In brief, DNA fragments had deoxyadenosine bases added to their 3′ ends, were ligated with paired-end adaptors, and amplified by PCR. The enriched libraries were sequenced with the Illumina NovaSeq 6000 platform and 150-bp paired-end reads were generated. The reads were aligned to the GRCh37/hg19 reference genome using BWA (http://bio-bwa.sourceforge.net/bwa.shtml). Picard tools (picard.sourceforge.net, version 1.46, MarkDuplicates) were applied to remove duplicates. The base quality of the reads was recalibrated using the GATK Haplotype Caller, which was also used to identify germline and somatic mutations and variations. Called SNV and InDel variants were annotated using the ANNOVAR package. FastQCv0.11.7 software was used to assess the sequencing data quality. Candidate somatic mutations were filtered based on international filtering criteria. Then, the filtered SNVs and InDels were screened with an alternative allele frequency <1% in public databases including the 1000 Genome Project and ESP 6500. Synonymous mutations were removed and deleterious mutations were retained with standard SIFT Score ≤ 0.05, PolyPhen-2 Sore ≥ 0.909, and Mutation Taster Score ≥ 0.85.

### Sanger Sequencing Validation

Shared non-synonymous SNVs and InDels identified in the two cancers were amplified using PCR Master Mix (Illumina, San Diego, CA, USA) and subjected to Sanger sequencing. Among the 20 mutation sites, five of them were close to another five target sites which could be amplified by the same primers. Therefore, we designed 15 primer pairs. Sequencing primers were designed using Primer3 (v.0.4.0) software and were generated by Sangon Biotech (Shanghai, China; Table 1).

**Table 1:** Primers for PCR in Sanger sequencing.

**Gene name**	**Location**	**Primer**	**Primer sequences (5′-3′)**	**Size**
				**(bp)**
MUC16	chr19:9050161	C1-F	GTTGTGATCATCATTTCTGTGGG	261
		C1-R	TAAGCTATTGCAAGTCCAGTAAGTG	
MUC16	chr19:9050163	C1-F	GTTGTGATCATCATTTCTGTGGG	261
		C1-R	TAAGCTATTGCAAGTCCAGTAAGTG	
MUC16	chr19:9058599	C2-F	TGGGTGGTGATGGTTATTTCTG	291
		C2-R	TGGCATTACAAGGATTGAGATAGA	
MUC16	chr19:9058600	C2-F	TGGGTGGTGATGGTTATTTCTG	291
		C2-R	TGGCATTACAAGGATTGAGATAGA	
MUC16	chr19:9065157	C3-F	GAATTTTCTCCGTATCTGTGGTG	243
		C3-R	CTCCATCTTCCCAGCTGTCTT	
MUC16	chr19:9076232	C4-F	CTCCAGAGCTCCTTGCCATT	304
		C4-R	CCCAGTAGTACAGAAGCAGAAGATG	
RPGRIP1L	chr16:53686476	C5-F	GGTAAAGAATGTTGTCCTTGCTC	412
		C5-R	TCTGGAGATAAAGAGCCTGTCAC	
RPGRIP1L	chr16:53686477	C5-F	GGTAAAGAATGTTGTCCTTGCTC	412
		C5-R	TCTGGAGATAAAGAGCCTGTCAC	
RPGRIP1L	chr16:53686660	C6-F	TGGACCTCAAGGGTGATAGTATTC	262
		C6-R	AAACCATCCACTTAGAACGAGG	
ERICH3	chr1:75037773	C7-F	GTCGTGATCTTTGGCTGCTAG	258
		C7-R	GAAAAGTCGCTGGAAAACATAAC	
ERICH3	chr1:75055676	C8-F	CTTTCGTCTATTGGCATCGG	428
		C8-R	AAAGATTGGGGACCACTGAGTAG	
TTN	chr2:179434170	C9-F	GTGATTTCTGTGACTTTCAGGTTAA	283
		C9-R	TAAATATAAGGGCAGGTGGCTC	
TTN	chr2:179500799	C10-F	GAAATCTCCCAGGAAAGTACTAACC	274
		C10-R	GAAGAGGAAGTCACAGTGGTCAA	
TTN	chr2:179640476	C11-F	GGACTTAGTTCAATCTTGTCAGGTT	289
		C11-R	TGAAGTTGAAAAGGGCTGAAAG	
RGSL1	chr1:182443294	C12-F	TATACAAATGCCTTCCCTGAAAATG	347
		C12-R	CCTGCCAGAGGTCAAGAAGTT	
RGSL1	chr1:182443296	C12-F	TATACAAATGCCTTCCCTGAAAATG	347
		C12-R	CCTGCCAGAGGTCAAGAAGTT	
RGSL1	chr1:182499947	C13-F	CCCAATTACCACTGAGAGTTCCT	250
		C13-R	ACTTTCTTCTTCACTAACCTGGG	
RGSL1	chr1:182499952	C13-F	CCCAATTACCACTGAGAGTTCCT	250
		C13-R	ACTTTCTTCTTCACTAACCTGGG	
OBSCN	chr1:228399569	C14-F	AAATAGGATGTGTGGAGGTGTTG	341
		C14-R	GCCAGGTCCAGGATAGTGAG	
OBSCN	chr1:228404900	C15-F	TTTGAGTGTGAGACCTCCGAAG	195
		C15-R	GAGCCGGAAGTCCACAGAGT	

### Immunohistochemistry

Samples of the patients' pancreatic adenocarcinoma and gingival squamous cell carcinoma were further analyzed by immunohistochemistry. Paraffin-embedded sections were deparaffinized and rehydrated, and sections were covered with Tris-EDTA (TE) buffer and heated for 10 min for antigen retrieval. Sections were incubated overnight at 4°C with an anti-RPGRIP1L antibody (1:200; 55160-1-AP, Proteintech, Wuhan, China), rinsed with phosphate buffered saline, and incubated with a secondary antibody (anti-rabbit) at 37°C for 30 min. The sections were finally incubated with 3,3′-diaminobenzidine and stained with hematoxylin. RPGRIP1L expression was identified by pathologists using microscopy.

### Bioinformatics Analysis

The Cancer Genome Atlas (TCGA) database was used to verify the expression of candidate genes in different cancers. The prognostic value of the hub genes was analyzed by TCGA, KM-plotter and an online tool called OncoLnc (http://www.oncolnc.org/). The pancreatic adenocarcinoma patients from the online database were divided into two groups at the optimal node based on the P value distribution curve. Co-expression analysis was performed with the condition of absolute correlation coefficient > 0.3 and *P* < 0.001. Gene-set enrichment analysis (GESA) and gene set variation analysis (GSVA) were conducted to identify possible signaling pathways, biological processes, and drug resistances related to the gene function. Statistical analysis was performed using R (version 3.6) and a *P*-value < 0.05 was considered significant.

### Protein Conformation Simulation

The RPGRIP1L structure information was obtained from the UniProt database (https://ebi10.uniprot.org/uniprot/Q68CZ1/), which showed existing crystal structure data obtained using the NMR method (PDB Entry: 2YRB, Chain: A, Positions: 595–737). The NMR structure of RPGRIP1L was acquired from the PDB database. The PRGRIP1L amino acid alterations were p.G708C and p.G708V. Protein conformation simulations were performed using the SWISS-MODEL online tool (https://swissmodel.expasy.org/). PyMOL software was used for structure visualization.

## Results

### Five Distinct Cancers Successively Developed in an Individual Patient

**Figure 1:**
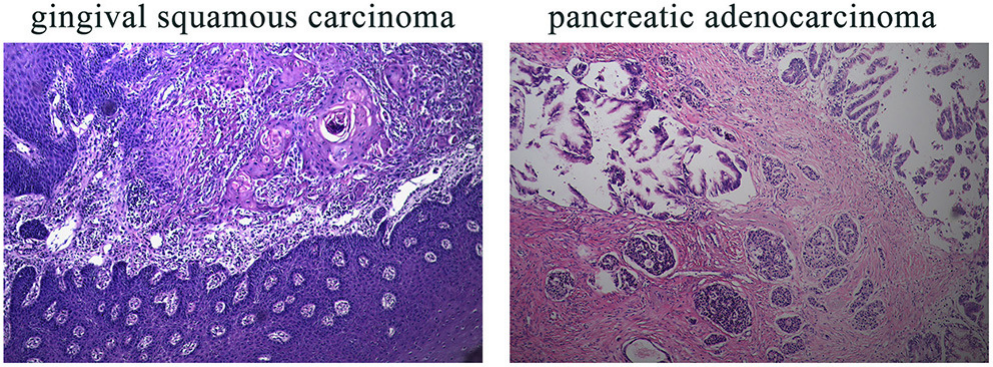
IHC results of the patient's gingival squamous carcinoma and pancreatic adenocarcinoma. IHC, immunohistochemistry. Magnification for all images: 20×.

This 78-year-old Chinese woman was first admitted to our hospital on November 2nd, 2019 with a right cervical mass. This mass was confirmed to be the result of metastasis of her previously diagnosed gingival squamous cell carcinoma. While taking her history, we discovered that she had a history of five different cancer types and a complicated family history. The five different cancer types were hepatocellular carcinoma in 2000, colorectal adenocarcinoma in 2002, invasive breast carcinoma in 2011, gingival squamous cell carcinoma in 2018, and pancreatic adenocarcinoma in 2018. Unfortunately, all of her cancers were treated in different hospitals and the first three cancer sample blocks were lost or damaged. Diagnosis of her first three cancers was based on her previous medical records and imaging examinations. The latter two cancer types, gingival squamous cell carcinoma and pancreatic adenocarcinoma, were confirmed by immunohistochemical methods (Figure 1).

### Candidate Variants Detected From WES

DNA extracted from the patient's samples was sequenced using WES. The results of her peripheral blood were applied to deduction of background variants. This identified 221 SNVs and InDels in her gingival squamous cell carcinoma and 281 SNVs and InDels in her pancreatic adenocarcinoma. Her peripheral blood results were used as a control. Comparison and analysis of the results revealed six common candidate genes with 20 mutation sites for further validation (Table 2).

**Table 2:** Summary of mutations analyzed by WES.

	**Gene ref gene**	**Chr**	**Start**	**End**	**Ref**	**Alt**
1	MUC16	19	9050161	9050161	-	AA
2	MUC16	19	9050163	9050163	-	CCACTTCCAGAGCC
3	MUC16	19	9058599	9058599	-	TGCC
4	MUC16	19	9058600	9058600	-	CTCCTGAGGTCTCCAG
5	MUC16	19	9065157	9065157	-	CTCACACCA
6	MUC16	19	9076232	9076232	-	ACTCTTGACTGGGACACTGGGAGATCTCTGTCAT
7	RPGRIP1L	16	53686476	53686476	C	A
8	RPGRIP1L	16	53686477	53686477	C	A
9	RPGRIP1L	16	53686660	53686660	-	AGACAACTCCCGTAGT
10	ERICH3	1	75037773	75037773	-	CCTGAAGGAAGGGCACCGCCAAGATGGAGAGGGGG
11	ERICH3	1	75055676	75055676	-	GACAGGGAAGCCCACACTGACA
12	TTN	2	179434170	179434170	-	A
13	TTN	2	179500799	179500799	C	G
14	TTN	2	179640476	179640476	G	C
15	RGSL1	1	182443294	182443294	-	TCTTGGAGGAGTG
16	RGSL1	1	182443296	182443296	-	TGACAATGGGGATGA
17	RGSL1	1	182499947	182499947	-	AA
18	RGSL1	1	182499952	182499952	-	GCTACTCGGGAGGCTGAGGCGAGTGGATCGCCTGAGGT
19	OBSCN	1	228399569	228399569	C	T
20	OBSCN	1	228404900	228404900	-	GGTGACTGTGGCTGCCACCAGCC

*WES, whole exome sequencing*.

### Validation of Mutation Sites by Sanger Sequencing

WES analysis identified a total of 20 variants in six genes, *ERICH3, OBSCN, RGSL1, TTN, RPGRIP1L*, and *MUC16*. These variants were subjected to confirmation using Sanger sequencing. Sanger Sequencing results confirmed two mutation sites in *RPGRIP1L*, one of *TTN*, and one of *OBSCN* in the patient's pancreatic adenocarcinoma, and two mutation sites in *TTN* in her gingival squamous cell carcinoma (Table 3).

**Table 3:** Summary of mutations verified by Sanger sequencing.

**Primer**	**Gene name**	**Location**	**Ref**	**Alt**	**YKD1489-A genotype**	**YKD1489-B genotype**
1	MUC16	chr19:9050161	-	AA	+/+	+/+
1	MUC16	chr19:9050163	-	CCACTTCCAGAGCC	+/+	+/+
2	MUC16	chr19:9058599	-	TGCC	+/+	+/+
2	MUC16	chr19:9058600	-	CTCCTGAGGTCTCCAG	+/+	+/+
3	MUC16	chr19:9065157	-	CTCACACCA	+/+	+/+
4	MUC16	chr19:9076232	-	ACTCTTGACTGGGACACTGGGAGATCTCTGTC	+/+	+/+
				AT		
5	RPGRIP1L	chr16:53686476	C	A	+/+	mut/+
5	RPGRIP1L	chr16:53686477	C	A	+/+	mut/+
6	RPGRIP1L	chr16:53686660	-	AGACAACTCCCGTAGT	+/+	+/+
7	ERICH3	chr1:75037773	-	CCTGAAGGAAGGGCACCGCCAAGATGGAGAG	+/+	+/+
				GGGG		
8	ERICH3	chr1:75055676	-	GACAGGGAAGCCCACACTGACA	+/+	+/+
9	TTN	chr2:179434170	-	A	+/+	mut/+
10	TTN	chr2:179500799	C	G	mut/+	+/+
11	TTN	chr2:179640476	G	C	mut/+	+/+
12	RGSL1	chr1:182443294	-	TCTTGGAGGAGTG	+/+	+/+
12	RGSL1	chr1:182443296	-	TGACAATGGGGATGA	+/+	+/+
13	RGSL1	chr1:182499947	-	AA	+/+	+/+
13	RGSL1	chr1:182499952	-	GCTACTCGGGAGGCTGAGGCGAGTGGATCGC	+/+	+/+
				CTGAGGT		
14	OBSCN	chr1:228399569	C	T	+/+	mut/+
15	OBSCN	chr1:228404900	-	GGTGACTGTGGCTGCCACCAGCC	+/+	+/+

*+/+, wild type; mut/+, mutant type. YKD1489-A, gingival squamous cell carcinoma; YKD1489-B, pancreatic adenocarcinoma*.

### Expression of the RPGRIP1L Protein in Cancers

**Figure 2:**
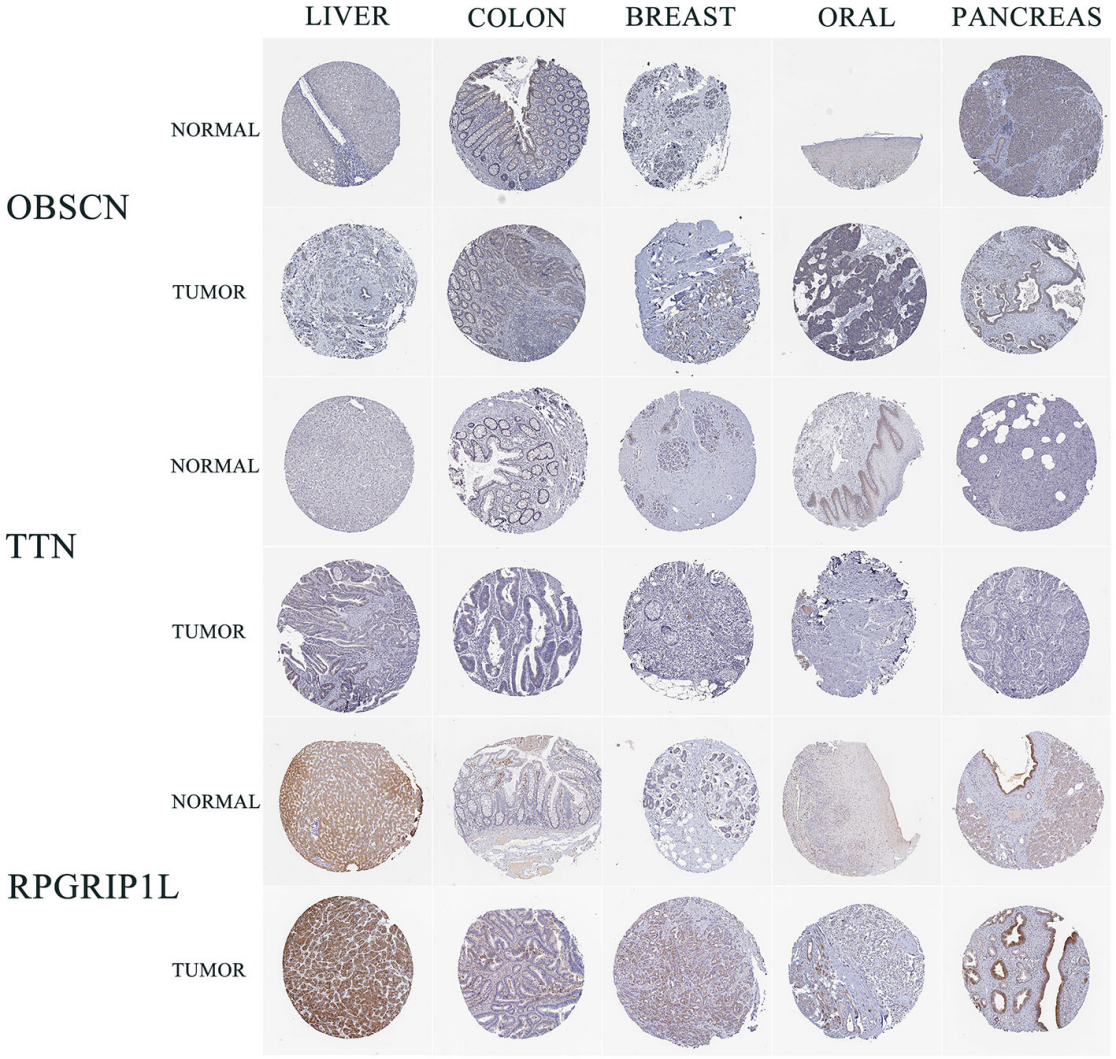
IHC results of three genes in five cancer types from HPA. IHC, immunohistochemistry; HPA, Human Protein Atlas; LIHC, liver hepatocellular carcinoma; COAD, colon adenocarcinoma; BRCA, breast invasive carcinoma; HNSC, head and neck squamous cell carcinoma; PAAD, pancreatic adenocarcinoma.

**Figure 3:**
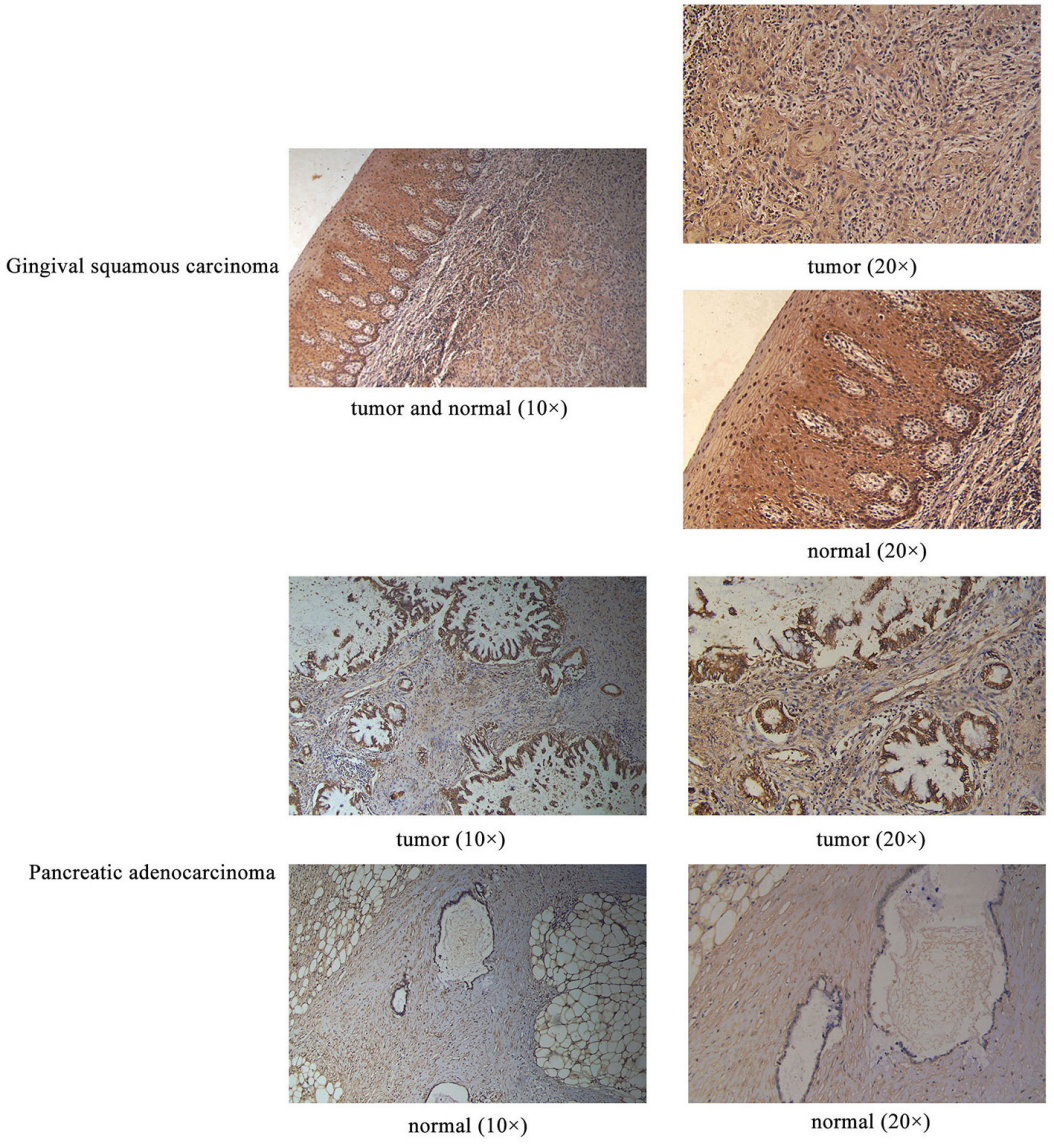
IHC results of RPGRIP1L protein expression in the patient's gingival squamous carcinoma and pancreatic adenocarcinoma. Magnification for all images: 20×.

**Figure 4:**
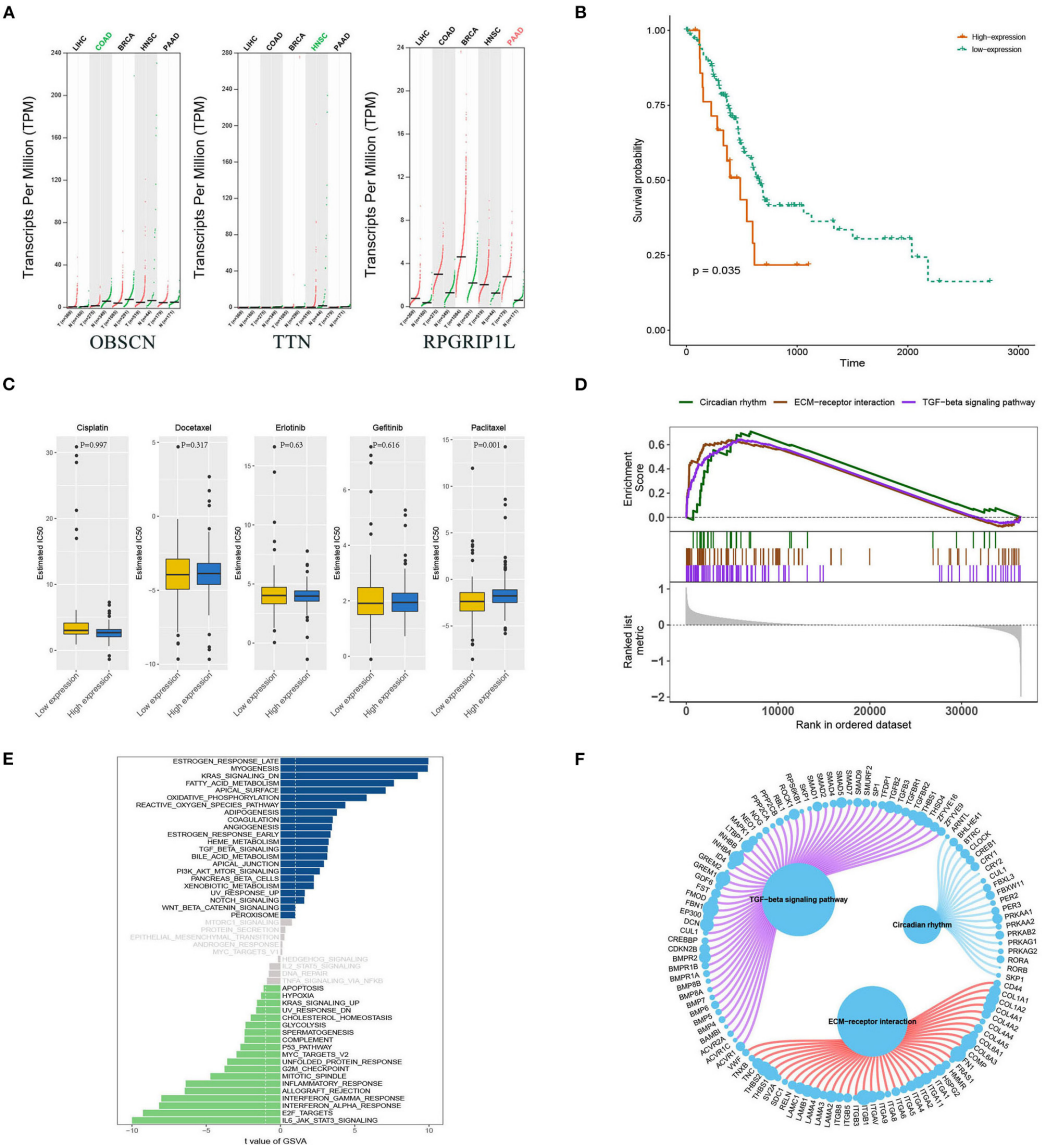
Bioinformatic analyses of RPGRIP1L gene expression and function. **(A)** Analysis of OBSCN, TTN, and RPGRIP1L mRNA expressions in five cancer types from TCGA database. The red and green colors of cancer types mean significantly difference (red, upregulated in tumor; green, downregulated in tumor, all compared with normal tissues). **(B)** The RPGRIP1L expression is related with PAAD patients' survival. **(C)** The RPGRIP1L expression is related with patients' sensitivity to paclitaxel. **(D)** GESA results showed that the RPGRIP1L expression was corelated with three pathways including circadian rhythm, ECM-receptor interaction, and TGF-β signaling pathways. **(E)** GSVA showed that RPGRIP1L expression was associated with several biological processes. Blue part means low expression, green part means high expression, gray part means no difference of RPGRIP1L expression. **(F)** GESA results of predicted RPGRIP1L binding targets in circadian rhythm, ECM-receptor interaction, and TGF-β signaling pathways. TCGA, The Cancer Genome Atlas; PAAD, pancreatic adenocarcinoma; GESA, gene-set enrichment analysis; GSVA, gene set variation analysis.

Sanger sequencing confirmed that RPGRIP1L, TTN, and OBSCN were mutated in this patient. We used the Human Protein Atlas (https://www.proteinatlas.org/) to analyze the level of protein expression from the three genes in multiple cancers. This analysis showed that RPGRIP1L is highly expressed in liver cancer, breast cancer, and pancreatic cancer, while expression of TTN and OBSCN was not markedly observed (Figure 2). The image sources are listed in Table 4. We also analyzed RPGRIP1L, TTN, and OBSCN mRNA expression using Gene Expression Profiling Interactive Analysis (GEPIA, http://gepia2.cancer-pku.cn/) tools. Results of this analysis were consistent with the observed protein expression results and showed that RPGRIP1L mRNA was more highly expressed than both TTN, and OBSCN mRNA in cancers (**Figure 4A**). Identification of high levels of RPGRIP1L expression in pancreatic cancer prompted us to extend this finding by analyzing RPGRIP1L protein expression in the patients' cancer samples. Immunostaining for RPGRIP1L in her pancreatic cancer tissues revealed predominantly cytoplasmic staining in most of the cancer cells, which demonstrated that RPGRIP1L protein expression was higher in her pancreatic adenocarcinoma tissues than adjacent pancreatic tissues, but was slightly lower in her gingival squamous cell carcinoma tissues than in normal gingival tissues (Figure 3).

**Table 4:** The sources of IHC pictures obtained from HPA.

**OBSCN (Antibody HPA065801)**
LIVER not detected: https://images.proteinatlas.org/65801/167889_A_7_4.jpg LIHC low: https://images.proteinatlas.org/65801/167888_B_9_3.jpg COLON not detected: https://images.proteinatlas.org/65801/167886_A_2_2.jpg COAD low: https://images.proteinatlas.org/65801/167886_A_2_2.jpg BREAST not detected: https://images.proteinatlas.org/65801/167889_B_2_4.jpg BRCA high: https://images.proteinatlas.org/65801/167886_A_6_8.jpg ORAL MUCOSA not detected: https://images.proteinatlas.org/65801/167889_A_8_1.jpg HNSC low: https://images.proteinatlas.org/65801/167887_A_2_8.jpg PANCREAS low: https://images.proteinatlas.org/65801/167889_A_1_3.jpg PAAD medium: https://images.proteinatlas.org/65801/167888_B_5_7.jpg
**TTN (Antibody HPA007042)**
LIVER not detected: https://images.proteinatlas.org/7042/165308_A_7_4.jpg LIHC medium: https://images.proteinatlas.org/7042/165306_B_9_7.jpg COLON not detected: https://images.proteinatlas.org/7042/165308_A_8_3.jpg COAD medium: https://images.proteinatlas.org/7042/165304_A_2_5.jpg BREAST not detected: https://images.proteinatlas.org/7042/165308_B_2_4.jpg BRCA not detected: https://images.proteinatlas.org/7042/165304_A_6_6.jpg ORAL MUCOSA low: https://images.proteinatlas.org/7042/165308_A_9_1.jpg HNSC medium: https://images.proteinatlas.org/7042/165328_A_2_6.jpg PANCREAS not detected: https://images.proteinatlas.org/7042/165308_A_3_3.jpg PAAD low: https://images.proteinatlas.org/7042/165306_B_5_2.jpg
**RPGRIP1L (Antibody HPA039405)**
PANCREAS medium: https://images.proteinatlas.org/39405/96534_A_2_3.jpg PAAD high: https://images.proteinatlas.org/39405/96535_B_6_7.jpg LIVER medium: https://images.proteinatlas.org/39405/96534_A_9_4.jpg LIHC high: https://images.proteinatlas.org/39405/96535_B_9_8.jpg COLON low: https://images.proteinatlas.org/39405/96534_A_8_3.jpg COAD low: https://images.proteinatlas.org/39405/96528_A_3_2.jpg BREAST low: https://images.proteinatlas.org/39405/96534_B_3_4.jpg BRCA high: https://images.proteinatlas.org/39405/96528_A_4_3.jpg ORAL MUCOSA not detected: https://images.proteinatlas.org/39405/96534_A_9_1.jpg HNSC low: https://images.proteinatlas.org/39405/96534_A_7_1.jpg

*IHC, immunohistochemistry; HPA, Human Protein Atlas; LIHC, liver hepatocellular carcinoma; COAD, colon adenocarcinoma; BRCA, breast invasive carcinoma; HNSC, head and neck squamous cell carcinoma; PAAD, pancreatic adenocarcinoma. The HNSC paired normal tissues are from human oral mucosa*.

### Bioinformatic Analyses Identify RPGRIP1L as a Potential Susceptibility Gene

**Figure 5:**
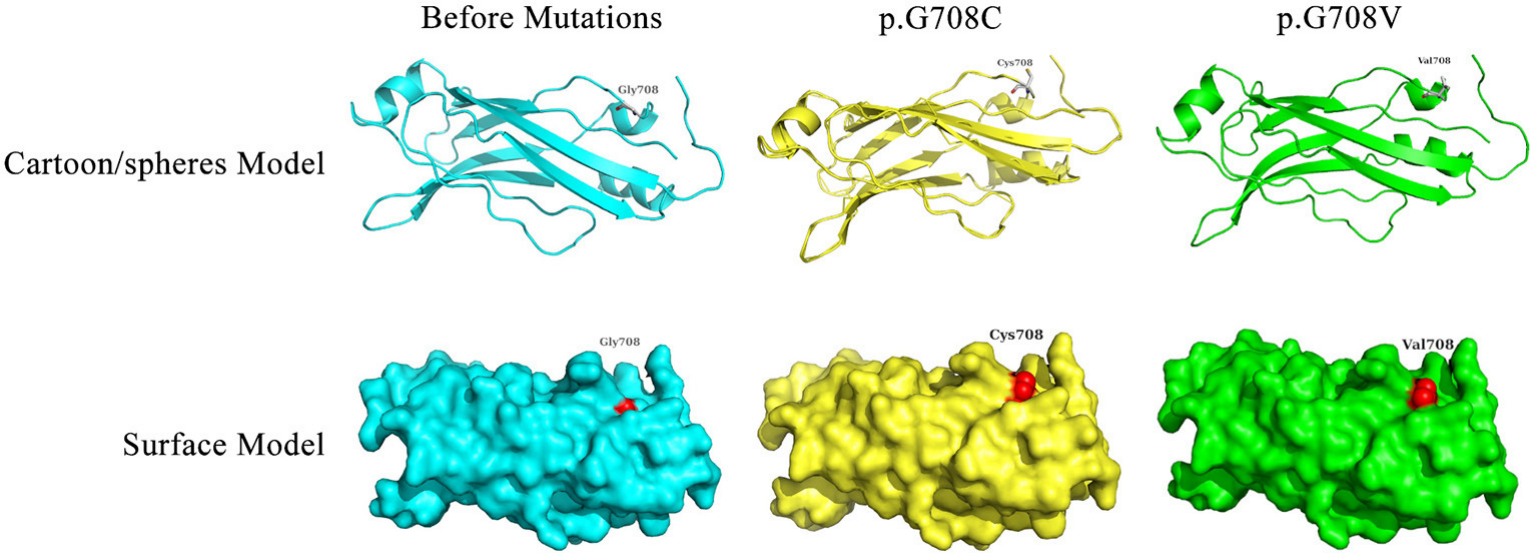
Display of the predicted protein structures before/after mutations of RPGRIP1L at position 708. The red color indicated the amino acid mutation site. Gly, glycine; Cys, cysteine; Val, valine.

Bioinformatics analyses were applied to explore the role of RPGRIP1L in pancreatic adenocarcinoma. In combination with the TCGA dataset, expression analysis revealed that *RPGRIP1L* mRNA was significantly upregulated in 179 pancreatic adenocarcinoma patients, (Figure 4A). The patients were then divided into two groups based on the minimum *P*-value of the curve. Using the Kaplan-Meier Plotter (KM, http://www.kmplot.com/) tool, survival analysis showed that *RPGRIP1L* expression was closely related to survival in patients with pancreatic adenocarcinoma (Figure 4B). Next, we performed GESA (Subramanian et al., 2005) and GSVA (Hanzelmann et al., 2013). The GESA results showed that *RPGRIP1L* expression corelated with three pathways: circadian rhythm, ECM-receptor interaction, and TGF-β signaling pathways (Figures 4D,F). GSVA showed that *RPGRIP1L* expression was associated with several biological processes including apoptosis, angiogenesis, and in part of classic signaling pathways involved in cancer development including the TGF-β, KRAS, PI3K-Akt-mTOR, p53, and Wnt/β-catenin signaling pathways (Figure 4E). As chemotherapy is commonly used to treat pancreatic adenocarcinoma, we estimated the chemotherapeutic response of patients with pancreatic adenocarcinoma using the R package “pRRophetic” (Geeleher et al., 2014). This analysis was based on the largest pharmacogenomics database GDSC (https://www.cancerrxgene.org/). This estimation was based on the half maximal inhibitory concentration (IC50), which revealed that *RPGRIP1L* expression was markedly associated with paclitaxel, one of the most widely used clinical anti-tumor drugs in cancer chemotherapy (Figure 4C). Protein conformation simulation was conducted using information from UniProt and PDB databases, and revealed that, in this patient, the glycine (Gly) at RPGRIP1L protein position 708 had changed to cysteine (Cys) or valine (Val) due to the somatic mutations in *RPGRIP1L*. The simulation demonstrated that these alterations changed the binding pocket structure from concave to convex. This could restrict contraction and extension, and interfere with the physiological function of the protein (Figure 5).

## Discussion

This study investigated the molecular alterations in a case of MPTs. RPGRIP1L was identified as a candidate gene with nonsynonymous SNVs (chr16:53686476, C>A; chr16:53686477, C>A) as a somatic mutation candidate. Moreover, bioinformatic analyses revealed that RPGRIP1L expression was related to patient' survival and chemosensitivity to paclitaxel in pancreatic adenocarcinoma, presumably occurring through the TGF-β signaling pathway.

Recently, great achievements have been made in the diagnosis and treatment of cancers. These advances have contributed to more satisfactory survival times and higher incidence of MPTs. It is generally accepted that inherited genetic defects are most likely to cause MPTs (Chan et al., 2018). A recent paper reported a rare variant within a *PARP4* pseudogene (PARP4P2) in a patient with MPTs and familial cancer history. They suggested that the PARP4P2 pseudogene variant could induce *PARP4* down-regulation, which might confer susceptibility to the development of multiple metachronous cancers (Cirello et al., 2019). In this study, our results represent newly discovered somatic mutations in the *RPGRIP1L* gene of a patient with MPTs and family history.

The retinitis pigmentosa GTPase regulator interacting protein 1-like (RPGRIP1L), also known as Ftm, localizes to the basal body-centrosome complex or to primary cilia and centrosomes in ciliated cells, is highly conserved (Wiegering et al., 2018). *RPGRIP1L* negative embryos show a variety of defects caused by cilia dysfunction (Vierkotten et al., 2007; Gerhardt et al., 2015). Defects in this gene can affect the development of several organs (Chen et al., 2015; Andreu-Cervera et al., 2019; Wang et al., 2019) and result in multiple diseases, including Joubert syndrome (JBTS) (Arts et al., 2007) and Meckel syndrome (MKS) (Delous et al., 2007). Previous studies revealed that RPGRIP1L plays an important role in the assembly of the transition zone, a region of the cilia (Jensen et al., 2015). *RPGRIP1L* was previously shown to govern the function of the Proteasome 26S Subunit, Non-ATPase 2 (PSMD2) by interacting with it and controlling the ciliary signaling through affecting ciliary proteasome activity (Gerhardt et al., 2015). Furthermore, it was recently demonstrated that *RPGRIP1L* deficiency impairs Hedgehog (Hh)/Gli signaling. RPGRIP1L can region-specifically influence mouse forebrain development through Hh signaling (Andreu-Cervera et al., 2019). RPGRIP1L was also reported to affect autophagy activity. Struchtrup et al. reported that absence of *RPGRIP1L* inhibits the initiation and later steps in the autophagy process, e.g., the generation of autophagosomes, which occurs *via* cilia-mediated mTOR signaling activation (Struchtrup et al., 2018). Recently, mutations in *RPGRIP1L* were reported in obesity and brain function. One study showed that rs13334070, in *RPGRIP1L* intron 4, has a significant association with obesity (Javanrouh et al., 2019). Reble et al. recently identified a SNV, rs7203525, that influences an alternative splicing event in *RPGRIP1L*, increasing exon 20 inclusion and potentially impacting brain function (Reble et al., 2020).

Using WES, our study discovered two novel RPGRIP1L SNVs in a case with MPTs, which were further validated by Sanger sequencing. Since the two SNVs were found only in the pancreatic adenocarcinoma tissues, and that the mutations occurred in exons, they are likely to influence translation efficiency, alternative splicing, and DNA copy number (El Marabti and Younis, 2018), and may be related to RPGRIP1L protein expression levels. However, the relationship between the SNVs and the observed expression differences needed to be validated further.

Previous studies had reported downregulation of RPGRIP1L in human hepatocellular carcinoma and suggested it was a tumor suppressor gene. Downregulation of RPGRIP1L increased Mitotic arrest deficient 2 (Mad2) protein levels, resulting in tumor cell transformation (Lin et al., 2009). Moreover, interaction between RPGRIP1L and Myosin Va, which was reported to be increased in several cancers, was detected at the ciliary base, indicating that RPGRIP1L might regulate the amount of Myosin Va and suppress tumorigenesis (Assis et al., 2017; Wiegering et al., 2018). Conversely, based on the above analysis and description, we contended that RPGRIP1L might act as a tumor promoter gene in pancreatic adenocarcinoma, since high RPGRIP1L expression was observed, and was closely related to patient's survival, TGF-β signaling pathway, and sensitivity to the chemotherapeutic drug paclitaxel. Additionally, protein conformation simulation analysis revealed that the identified mutations might affect the binding ability of the protein and impact downstream targets. In combination with the previous studies, our results suggest that RPGRIP1L might play a promoter or suppresser role in a cancer type-specific manner.

Finally, our study has some limitations. Samples of the patient's other three cancer tissues were not available, and the novel *RPGRIP1L* mutations were not found in gingival squamous cell carcinoma. One conceivable explanation is that her gingival squamous cell carcinoma might be caused by dietary habit and lack of oral care. Indeed, people from this region have a higher incidence of esophageal squamous cell carcinoma because of their preference for pickled and hot foods. The patient also suffered from diabetes, which appeared to be linked to oral cancer (Mekala et al., 2020). Moreover, samples from her family members were not available as well. Due to her complicated family history of malignancy, we could not exclude the possibility that there were germline mutations inherited by the patient. Further investigations are required to clarify the potential role of *RPGRIP1L* mutations in tumorigenesis and its value as a therapeutic target.

## Data Availability Statement

The raw data supporting the conclusions of this article will be made available by the authors, without undue reservation.

## Ethics Statement

The studies involving human participants were reviewed and approved by Clinical Research Ethics Committee of The First Affiliated Hospital of University of Science and Technology of China. The patients/participants provided their written informed consent to participate in this study.

## Author Contributions

MH and XS designed the research. JG and YY performed the experiments. ZJ provided the clinical samples. ZJ, MY, and XX analyzed and interpreted the data. JG wrote the manuscript. MH critically commented and edited the manuscript. All authors read and approved the final version of the manuscript.

## Conflict of Interest

The authors declare that the research was conducted in the absence of any commercial or financial relationships that could be construed as a potential conflict of interest.

## Supplementary Material

Supplementary Figure 1 The pedigree tree of the family.
