# Supplementary figures and images for: Case Report: Novel RPGRIP1L Gene Mutations Identified by Whole Exome Sequencing in a Patient With Multiple Primary Tumors
Source: Front Genet. 2021 Feb 1;12:620472. doi: 10.3389/fgene.2021.620472 (PMC7882720; doi:10.3389/fgene.2021.620472)

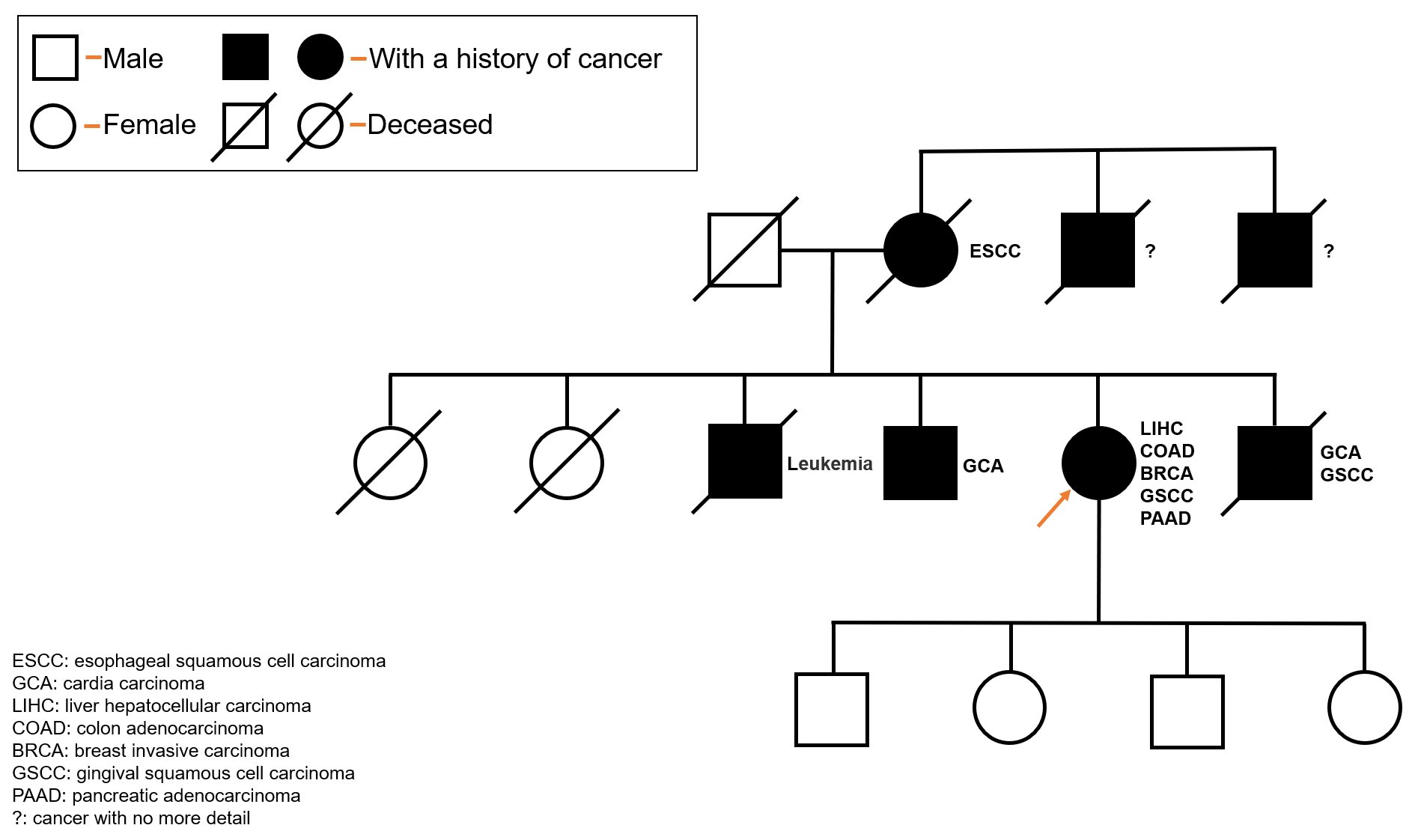

Supplement: Supplementary Figure 1 — The pedigree tree of the family. [file Image_1.TIF]
